# A Fenton Oxidation-Based Integrated Strategy for the Treatment of Raw Gasoline Alkali Residue in Kashi

**DOI:** 10.3390/toxics13100871

**Published:** 2025-10-13

**Authors:** Yucai Zhang, Xianghao Zha, Zhuo Zhang, Yangyang Guo, Shuying Yang, Haonan Qiu, Zhiwei Li

**Affiliations:** 1 Xinjiang Key Laboratory of Novel Functional Materials Chemistry, College of Chemistry and Environmental Sciences, Kashi University, Kashi 844000, China; 2 Kezilsu Ecological Environment Monitoring Station, Atushi 845350, China; 3 State Key Laboratory of Regional and Urban Ecology, Institute of Urban Environment, Chinese Academy of Sciences, Xiamen 361021, China

**Keywords:** gasoline alkali residue, fenton’s reagent, chemical oxygen demand, wastewater treatment, advanced oxidation technology

## Abstract

Gasoline alkali residue raw liquid, a kind of highly toxicity containing organic waste generated during petroleum refining, is characterized by its complex composition, high pollutant levels, and significant emission volume. The effective treatment of this wastewater remains a considerable challenge in environmental engineering. This study systematically investigates the degradation efficiency and mechanism of Fenton oxidation in reducing the chemical oxygen demand (COD) of raw gasoline alkali residue sourced from Kashi. The effects of H_2_O_2_ concentration and the H_2_O_2_/Fe^2+^ molar ratio on COD and TOC removal were examined. Results demonstrated that the COD and TOC removal efficiency exhibited an initial decrease followed by an increase with rising concentrations of Fe^2+^ and H_2_O_2_. Comparative assessment of different combined Fenton processes revealed distinct mechanistic differences among the composite oxidation systems. The integration of pretreatment with UV-Fenton oxidation was identified as the optimal strategy. Under optimal conditions (pH = 3.0, H_2_O_2_ concentration = 1.0 mol/L, H_2_O_2_/Fe^2+^ molar ratio = 5:0.10), the COD was reduced from 25,041 mg/L to 543 mg/L, achieving a COD removal rate of 97.8%. This study elucidates the reaction mechanism of the Fenton system in treating alkali residue and provides a theoretical foundation for the advanced treatment of high-concentration organic wastewater.

## 1. Introduction

### 1.1. Sources and Properties of the Gasoline Alkali Residue Stock Solution

Environmental protection has received great attention since China’s sustainable development strategy has been implemented, and the harmless treatment of waste generated during industrial production is particularly important [1,2]. Additionally, the treatment of several alkali residue stock solutions generated in petroleum refining is imminent [3]. Refinery alkali residue crude liquid primarily comes from the petrochemical industry refinery catalytic cracking gasoline alkaline wash, total hydrocarbon alkaline wash crude liquid, and alkali residue crude liquid produced by the atmospheric decompression device [4]. These raw alkali residues contain several sulfides, mercaptans, phenols, thiophenols, thioethers, naphthenic acids, and other toxic and hazardous pollutants. Moreover, these raw alkali residues have pH values between 10 and 14, are usually black–brown in color with phenols featuring a high amount of volatile phenols and a strong, irritating odor [5,6].

### 1.2. Treatment Mode of Gasoline Alkali Residue Crude Fluid

Due to the strong alkalinity, high toxicity, and difficult biochemical degradation characteristics of gasoline alkali residue crude fluid, its efficient treatment is a critical problem in petroleum refining. Usually, the recovery of naphthenic acid, extraction, low-temperature wet oxidation, and other technologies are used to treat the crude liquid in petroleum refineries [7]. However, the final treatment indicators often do not meet the relevant technical and economic requirements. Common treatment methods include incineration [8], membrane separation [9,10], and biochemical methods (anaerobic and aerobic) [11]. Although these methods can effectively treat waste liquids, they require large investments, expensive operating costs, and are susceptible to external conditions. Consequently, the investigation of novel alkali residue raw liquid deep treatment technology is a key study field in industrial raw liquid purification and treatment technology [12]. Using redox treatment technology, the organic matter in the raw liquid is decomposed using an oxidizer and a catalyst at high temperatures and pressures [13]. Subsequently, this method has the advantages of high degradation efficiency and less secondary pollution. The common redox methods are categorized into wet oxidation (WAO), supercritical wet oxidation (SWAO), and others [14,15].

In the Fenton system [16], Fe^2+^ and H_2_O_2_ primarily act as the homogeneous catalyst and the oxidizing agent, respectively [17]. Fenton reagent is the primary choice for the deep oxidation of industrial stock solutions (AOP) due to its unique advantages in the oxidative degradation of POPs [18,19,20]. In the ordinary Fenton reagent treatment of stock solutions, H_2_O_2_ decomposed under the catalytic effect of Fe^2+^ to produce hydroxyl radicals (**·**OH), oxidizing the organic matter into small molecules through electron transfer. Simultaneously, O_2_ finally oxidized the catalyst Fe^2+^ to Fe^3+^, and the resulting Fe(OH)_3_ colloid has a good flocculation effect under certain conditions, removing several suspended particles in the waste liquid [21,22]. Ordinary Fenton reagents can oxidize organic molecules under dark conditions, offering the advantages of low investment and easy operation. However, the intermediate products of the reaction transformation or the formation of complexes with Fe^3+^ that compete with **·**OH generation may cause more harm to the environment [13].

The traditional wet oxidation method uses an oxidizing agent to oxidize the organic matter in the raw liquid to CO_2_ and H_2_O under high temperatures and pressures to remove pollutants. The photocatalytic oxidation method is a typical deep oxidation technology and is the degradation treatment of organic stock solutions using a specific light source and catalyst compound [23]. Additionally, its mechanism is that the light energy absorbed by the photosensitized semiconductor (catalyst) is higher than the energy of its forbidden bandwidth, producing free electrons and holes. The reaction between holes and water and between electrons and dissolved oxygen produces **·**OH and O^2−^, respectively. Additionally, **·**OH and O^2−^ have strong oxidation properties to promote the degradation of organic matter that is difficult to adsorb. The raw liquid has an effectively reduced COD value after adsorption treatment when the Fenton reagent catalytic oxidation is added. Compared with conventional treatment technology, deep oxidation technology has the advantages of a wide range of use, high treatment efficiency, fast oxidation speed, and less secondary pollution [24].

### 1.3. Fenton Reagent Treatment of Gasoline Alkali Residue Stock Solution from Kashi Refinery

Kashi petroleum refinery produces about 50,000 tons of alkali residue stock solution per year during the refining years, which are primarily concentrated in stock solution pools around Kashi. The treatment of the raw slag generated by the Kashi petrochemical plant is extremely urgent since its high alkalinity and long-term accumulation subject the protective layer at the bottom of the pool to the dangers of leakage and infiltration. Based on the traditional wet oxidation method, the organic matter in the raw solution generally has a high COD value (above 20,000 mg/L), and the degradation requires 250–350 °C high temperature and 30–80 MPa pressure, which increases the cost of treatment. Although the Fenton oxidation system has been widely used to treat gasoline alkali residue, it has shortcomings, like low treatment efficiency, slow oxidation rate, limited scope of use, and high costs [25]. To overcome these limitations, the low-cost and sustainable operation of the composite Fenton oxidation must be developed.

After a series of orthogonal tests and one-way tests, optimal process parameters were preferred due to the complexity of the raw alkali slag produced by Kashi petroleum refinery. The process, consisting of pretreatment and Fenton oxidizer, could significantly reduce the COD and TOC removal of the effluent of the raw alkali slag. After several organic substances in the raw slag were adsorbed and treated, the organic substances in the raw slag were further degraded using visible light-Fenton oxidation, ozone-Fenton oxidation, UV-Fenton oxidation. The composite Fenton oxidation technology could significantly reduce the requirements of the treatment equipment while reducing the oxidizer consumption. Consequently, using this process, the raw lye residue of the petrochemical plant reached the sewage discharge standard in Kashi (COD < 1000 mg/L “Petroleum Refining Industry Pollutant Discharge Standard”), and the treated raw liquid could be recycled or discharged into the plant. This waste liquid degradation treatment program has a wide range of applications and is a reference for treating high-concentration organic raw liquids generated by most petrochemical enterprises.

## 2. Experimental Section

### 2.1. Pretreatment of the Alkali Residue Liquid

**Figure 1:**
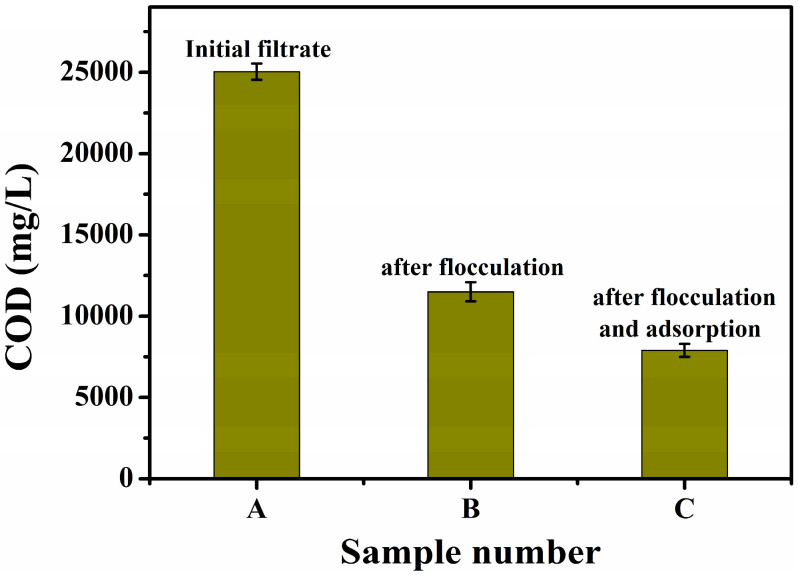
COD values of the effluents of different filtrates of the alkali slag after pretreatment.

Alkali residue liquid (500 mL) from Kashi petrochemical refinery was taken, and 0.1% polyacrylamide flocculant for sewage was added to the filtrate A (COD = 25,041 mg/L) after filtration with 30 min of stirring. Heavy snowflake precipitation appeared, with an obvious effect of flocculation. After flocculation of the liquid B (COD = 11,500 mg/L), after filtration by adding 5% of activated carbon sewage adsorbent, it was stirred for 30 min and filtered to obtain filtrate C (COD = 7898 mg/L). Prior to the addition of Fenton reagents (FeSO_4_·7H_2_O and H_2_O_2_), the alkaline wastewater was actively acidified to the optimal pH of 3.0 using a suitable acid (H_2_SO_4_) and NaHCO_3_ solution. A COD meter (GDYS-101SQ3) was used to determine the COD values (the average of three replicate tests) of the three filtrates, and the results are shown in Figure 1. Compared to the effluent from the filtrate after flocculation and adsorption, the alkali residue in the original solution of the effluent had a significantly higher COD value, indicating that it contained more organic pollutants. The effluents from the filtrate after flocculation and adsorption had significantly reduced COD values, implying that a part of the organic components had been processed, but proper purification was needed to meet the discharge standard.

### 2.2. Orthogonal Test to Optimize the Fenton Oxidation Conditions

(i) Optimization of the H_2_O_2_ concentration: As seen in Figure 1, the COD value of the effluent of the original liquid after pretreatment could be reduced to about 8000 mg/L. The amount of H_2_O_2_ was changed according to the proportion shown in Table 1 to investigate the effect of the H_2_O_2_ concentration on the removal of the organic substances in the stock solution in Fenton oxidation. After this treatment, the nine samples were placed at room temperature and atmospheric pressure for 4 h, followed by measuring the COD value of the effluent of each test solution using a COD meter for comparison.

(ii) Optimization of the H_2_O_2_ and Fe^2+^ (FeSO_4_·7H_2_O) ratios: The pH value of the test solution was adjusted to 3 using a calibrated pH meter (PHG-21C), and the H_2_O_2_ and Fe^2+^ contents were determined by inductively coupled plasma mass spectrometry (ICP-MS) proportionally according to Table 2. The 25 samples with different H_2_O_2_ to Fe^2+^ mass ratios (m(H_2_O_2_):m(Fe^2+^)) were placed at room temperature and atmospheric pressure for 4 h. The COD values of the test liquids were determined using a COD meter for comparison.

### 2.3. Comparison of the Composite Fenton Oxidizing Treatment Methods

First, 500 mL of raw alkali slag was taken from Kashi Petrochemical Plant, followed by the addition of 0.1% composite flocculant special for sewage into the filtrate after filtration, stirring for 30 min on a heating jacket magnetic stirrer (SH05-3T), and drawing the filtrate. Then, 5% of the special adsorbent for sewage was added, followed by 30 min of stirring and filtration to pre-treat the test solution. Then, 90 mL of the test solution was placed in three 150 mL beakers, which were treated and compared using the following three composite methods.

(1) Visible light–Fenton method

H_2_O_2_ and Fe^2+^ were added to the test solution according to the H_2_O_2_ and Fe^2+^ injection ratio of 2:0.10. The test solution turned dark black in color, and tiny bubbles were released. Then, the test solution was kept in the sunlight for 4 h. Consequently, the solution was clarified, and it turned light yellow in color. The test solution was labeled as I, and the COD value of its effluent was measured using a COD meter.

(2) UV–Fenton method

H_2_O_2_ and Fe^2+^ were added to the test solution according to the H_2_O_2_ to Fe^2+^ ratio of 2:0.10. The test solution turned dark black in color, and tiny bubbles (CO_2_) were released. Then, the test solution was placed under a GPH single-ended four-pin quartz ultraviolet germicidal lamp irradiation with stirring for 4 h. Consequently, the test solution was clarified, but its light yellow color was slightly darker compared to that of test solution I. The test solution was labeled as II, and its COD value was measured using a COD meter.

(3) Ozone–Fenton method

H_2_O_2_ and Fe^2+^ were added to the test solution according to the H_2_O_2_ and Fe^2+^ injection ratio of 2:0.10. The test solution turned black, and was accompanied by the release of small bubbles (CO_2_). When O_3_ was passed through the solution for 1 h, its color changed to brown. The test solution was labeled as III, and its COD value was measured using a COD meter. Furthermore, test solution III was kept under the sunlight for 4 h, and its color was light yellow and clarified. This test solution was labeled as IV, and its COD value was measured using a COD meter.

## 3. Results and Discussion

### 3.1. Optimization of the Fenton Oxidation Conditions

**Figure 2:**
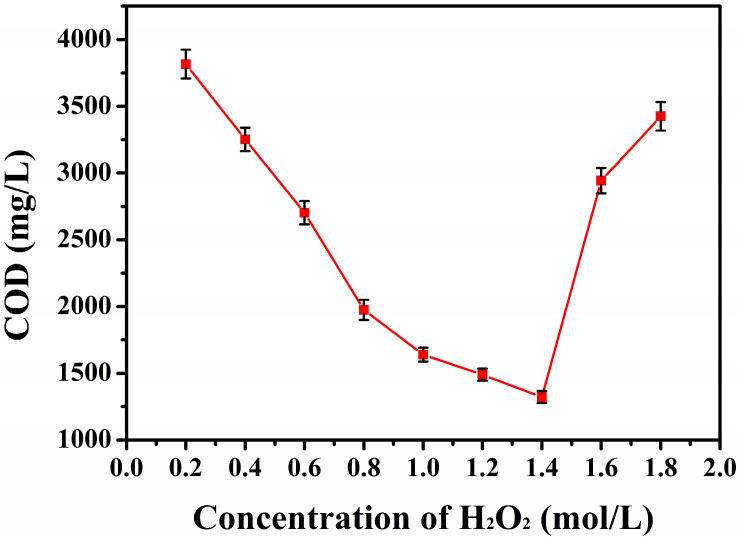
COD variation curves of the test solutions with different H_2_O_2_ contents.

During Fenton oxidation, the rate of **·**OH generation was positively correlated with the concentration of the added H_2_O_2_. As shown in Figure 2, the COD value of the test solution first decreased and then increased as the H_2_O_2_ concentration increased. The COD value fell to the lowest value when the H_2_O_2_ concentration was 1.4 mol/L. The COD value started to increase gradually when the H_2_O_2_ concentration exceeded 1.4 mol/L. This was because the rate of **·**OH generation was higher at higher H_2_O_2_ concentrations, and the COD value of the test solution decreased significantly under the action of **·**OH. When the H_2_O_2_ concentration was too high, the excess H_2_O_2_ could not decompose to produce more **·**OH, and Fe^2+^ was oxidized to Fe^3+^ at the beginning of the rapid oxidation, which decreased the COD value of the test solution to the lowest. Instead, Fe^2+^ was rapidly oxidized to Fe^3+^ at the beginning of oxidation, and Fe^3+^ catalyzed the oxidation process to consume H_2_O_2_ and reduce the **·**OH production. Therefore, the optimized H_2_O_2_ concentration in this process was 1.4 mol/L.

**Figure 3:**
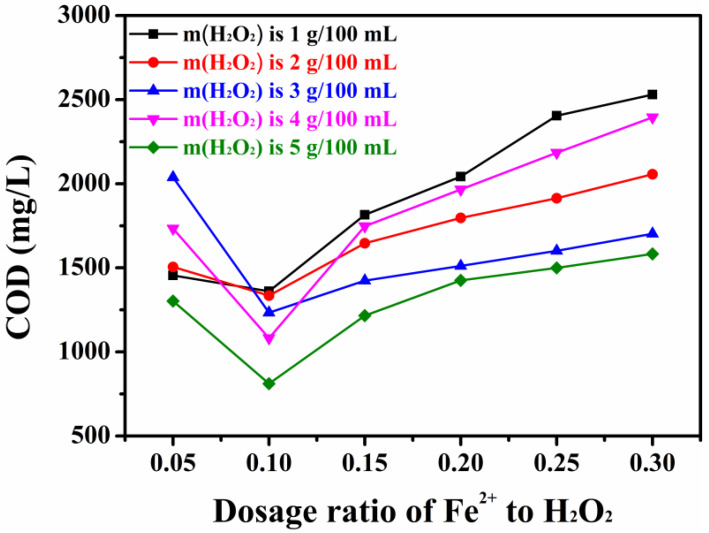
Effect of the H_2_O_2_ to Fe^2+^ ratio on the COD value.

As seen in Figure 3, the COD of the test solution gradually increased as the mass ratio of the added H_2_O_2_ increased. The 810 mg/L COD value of the test solution at H_2_O_2_ and Fe^2+^ mass ratios of 5 g/100 mL and 0.10 g/100 mL, respectively, was the best COD and TOC removal efficiency in this case. Considering the production cost, using H_2_O_2_ at a mass ratio exceeding 5 g/100 mL was not economically viable.

**Figure 4:**
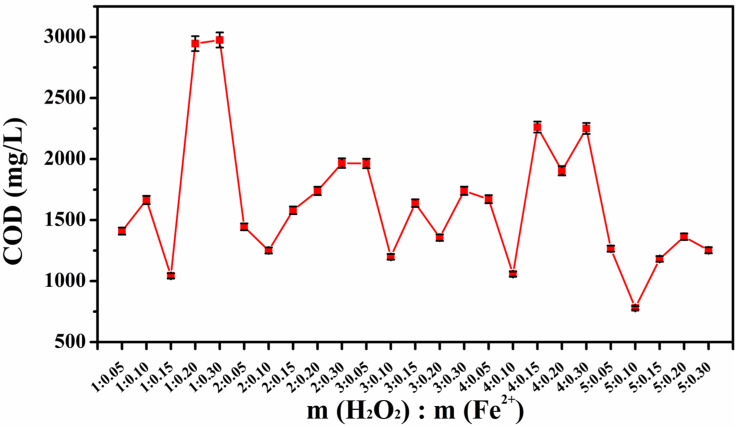
COD values of the test solutions with different preferred H_2_O_2_ and Fe^2+^ mass ratio dosages in the orthogonal test.

As shown in Figure 4, the COD values had a regular trend for the test solutions with different values of m(H_2_O_2_):m(Fe^2+^). According to the synergistic oxidation characteristics of organic matter using H_2_O_2_ and Fe^2+^ in the Fenton reaction, the COD value was high for low H_2_O_2_ ratios for the excess of Fe^2+^ led to the rapid consumption of H_2_O_2_, and the generated **·**OH was insufficient for the organic matter degradation. In contrast, the excess H_2_O_2_ for excessive H_2_O_2_ ratios may have a side reaction with **·**OH (H_2_O_2_ + **·**OH → H_2_O + HO_2_**·**) to reduce the oxidizing efficiency and slow the decrease in the trend of COD. When the H_2_O_2_ and Fe^2+^ ratios were close to the optimal conditions and the **·**OH generation rate matched the demands of the organic matter degradation, the reduced COD could be close to the lowest value. The reduction in COD was the most obvious when m(H_2_O_2_):m(Fe^2+^) was 5:0.10. Therefore, to achieve a better removal of organic matter in the stock solution, the optimal m(H_2_O_2_):m(Fe^2+^) determined was 5:0.10.

**Figure 5:**
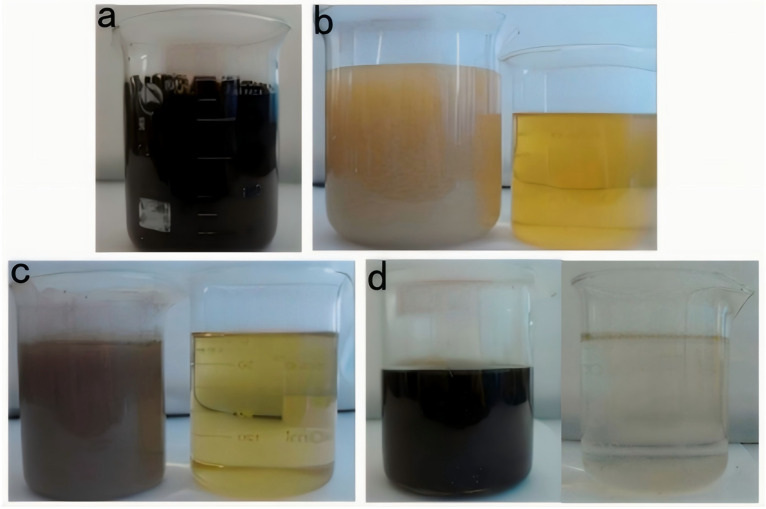
Photographs of raw alkali slag after different treatment stages. (**a**) Raw alkali slag, (**b**) test solution after flocculation, (**c**) test solution after adsorption, and (**d**) test solution after oxidation by adding Fenton’s reagent.

**Figure 6:**
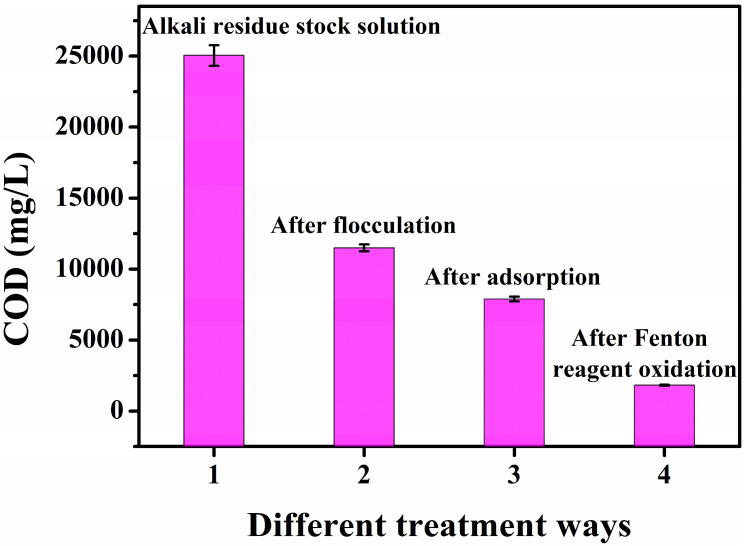
Changes in the COD values of the alkali slag after different treatment stages.

As seen in Figure 5, the raw alkali residue was a black-brown colored turbid liquid, with numerous suspended particles. Additionally, the stratification phenomenon could be seen when it was static. A thick gray–black solid slag was deposited at the bottom, and the low transparency of the liquid in the upper layer indicated a high concentration of organic pollutants and inorganic suspended solids in the original liquid. The high alkalinity of the alkali slag (pH > 13) could be the primary reason for its dark color. After adding the polyacrylamide flocculant, the test solution showed obvious flocculation. At the initial stage, the stock solution changed its color from dark to light brown, and the suspended particles rapidly aggregated to form white or off-white flocs, accompanied by volume expansion (Figure 5b). As the stirring speed was gradually reduced, the flocs gradually sank and the upper layer of the liquid had a significantly improved transparency. Additionally, the charge neutralization effect aggregated the colloidal particles at this stage to ultimately form flocs that could settle down. After treatment with the diatomaceous earth adsorbent, the color of the test solution further lightened to pale yellow (Figure 5c). Numerous dark-colored pollutants attached to the adsorbent surface to form a black granular precipitate, and the suspended matter in the liquid almost completely disappeared. The adsorption process selectively removed the pigments, heavy metal ions, and small organic molecules from the stock solution using physical adsorption and chemical bonding. For the initial phase of the reaction (within the first 10 min), despite a high phenol removal efficiency exceeding 80%, the acute toxicity of the effluent, as assessed by the inhibition of luminescent bacteria, increases significantly. This initial rise in toxicity is attributed to the generation of transient, yet highly toxic, aromatic intermediates. GC-MS analysis confirmed the presence of key compounds such as p-benzoquinone and catechol (o-dihydroxybenzene), which are known to be more toxic than phenol itself. These intermediates exert their toxic effects through mechanisms like oxidative stress and the formation of protein adducts [26]. Only upon prolonged reaction time (120 min) are these hazardous intermediates effectively mineralized into low-molecular-weight organic acids and ultimately CO_2_ and H_2_O, leading to a subsequent significant decrease in the overall toxicity of the treated effluent. After adding Fenton’s reagent (Fe^2+^/H_2_O_2_), the test solution initially turned orange–red (the color of hydrated Fe^3+^), followed by the color gradually fading with oxidation and finally turning light yellow or colorless. The reaction process was accompanied by the generation of bubbles (O_2_ release), and reddish-brown flocculent (Fe(OH)_3_) precipitates appeared at the bottom. The Fe(OH)_3_ sludge was acidified with H_2_SO_4_, which effectively re-dissolved the iron, producing a Fe_2_(SO_4_)_3_ solution. At this stage, the strong oxidizing effect of **·**OH decomposed the hard-to-degrade organic matter into CO_2_ and H_2_O, while Fe^2+^ was oxidized to Fe^3+^, and Fe(OH)_3_ precipitate could be converted into Fe_2_(SO_4_)_3_. As seen in Figure 6, flocculation, adsorption, and Fenton oxidation significantly reduced the COD value of raw alkali residue liquid, indicating that the process could effectively remove the organic substances in the raw liquid.

### 3.2. Oxidation Mechanism of the Fenton Reaction

#### 3.2.1. Analysis of the Fenton Oxidation Mechanism

The Fenton reagent had a strong oxidizing ability because the system contained Fe^2+^ and H_2_O_2_, Fe^2+^ catalyzed H_2_O_2_ to generate **·**OH, and **·**OH had a stronger redox potential compared with other oxidants [27]. As seen in Table 3, the pH value significantly influenced the Fenton system, and a pH value that was too high or too low was not conducive to generating **·**OH. An excessively high pH value would inhibit the content of **·**OH generated. The reduction of Fe^3+^ to Fe^2+^ was difficult when the pH value was too low, and the insufficient supply of Fe^2+^ was not conducive to generating **·**OH. The H_2_O_2_ and Fe^2+^ contents significantly affected the generation of **·**OH. When the H_2_O_2_ and Fe^2+^ contents were low, the generated **·**OH content was relatively small. At the same time, H_2_O_2_ was the capture agent of **·**OH, and a too high H_2_O_2_ dosage could cause the initial generation of **·**OH to be lost. Additionally, too high Fe^2+^ dosage could generate a large content of reactive **·**OH in H_2_O_2_ at the beginning of the reaction under high catalyst concentration. Additionally, the restricted reaction between **·**OH and the substrate led to the accumulation of the unconsumed free **·**OH and reaction of **·**OH with each other to generate water, resulting in the partial consumption of the initially generated **·**OH [28]. Therefore, a too high Fe^2+^ dosage was not conducive to generating **·**OH, and the Fe^2+^ to H_2_O_2_ dosage ratio was to be strictly controlled in practical applications.

#### 3.2.2. The Composite Fenton Oxidation System Mechanism

**Figure 7:**
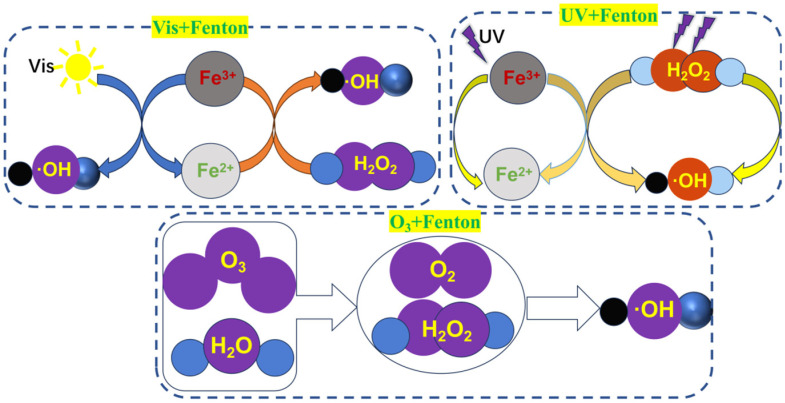
Schematic diagram of the mechanism of different composite Fenton oxidation treatments.

Numerous studies are exploring the use of inexpensive materials to improve the Fenton oxidation system to reduce its processing cost (Figure 7). The common composite Fenton oxidation systems are categorized into the following:

(1) Visible light–Fenton method

Light could significantly improve the degradation efficiency in the oxidation process of Fenton reagents. The visible light-assisted Fenton oxidation system is known as the visible light–Fenton method (Vis–Fenton method). The results of the oxidative degradation of the phenol stock solution using the Vis–Fenton method showed that the suitable pH value for the reaction was between 3 and 4. Additionally, the participation of moderate Fe^2+^ contents in the Vis–Fenton method could help phenol degradation, but an excessive Fe^2+^ content was not conducive to phenol degradation [29]. The visible light–Fenton oxidation could increase the COD/TOC ratio from 0.10 to 0.32 and improve the biochemical degree.

(2) Ultraviolet–Fenton (UV–Fenton)

Compared to the Vis–Fenton method, the UV–Fenton oxidation method had a stronger ability to treat the stock solution due to the synergistic effect of UV light and the catalytic effect of Fe^2+^ on H_2_O_2_. Additionally, the method could reduce the Fe^2+^ dosage and increase the H_2_O_2_ utilization rate. The results showed that oxalate and citrate introduced into the UV–Fenton system could effectively improve the utilization of UV light resulting in the UV/ferric oxalate complex-H_2_O_2_ method, where the ferric oxalate complex strongly absorbed UV light, and the quantum yield under visible light irradiation could reach 1.0–1.2, so that the Fenton reagent can continue to be provided [30]. The UV/iron oxalate complex-H_2_O_2_ method was an extension of the UV–Fenton method, which improved the energy utilization and saved the use of H_2_O_2_.

(3) Ozone-Fenton (O_3_-Fenton)

Compared to the ordinary Fenton method, the Vis–Fenton method could effectively improve the mineralization of organic matter. However, it had the disadvantages of low photoefficiency and an imperfect automatic H_2_O_2_ generation mechanism. The essence of the O_3_–Fenton method was to combine the Fenton oxidation treatment and O_3_ oxidation. It could degrade more types of organic matter, in addition to processes, including **·**OH oxidation and O_3_-enhanced oxidation [31]. Presently, there are not many reports on adding O_3_ in the oxidation process of the Fenton reagent, and the O_3_–Fenton method can become a major research hotspot for the Fenton oxidation technology, considering the development history of the Fenton reagent.

### 3.3. Treatment Results of the Composite Fenton Oxidation System

**Figure 8:**
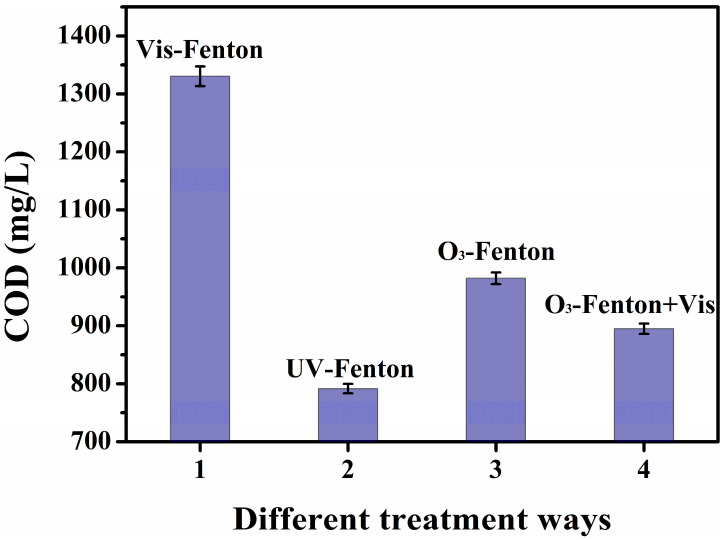
COD values of different composite Fenton oxidation systems after treating the alkali slag stock solution.

As seen in Figure 8, the raw alkali slag treated with different composite Fenton oxidation systems had different COD values. Compared with the original liquid, the Vis-Fenton method could reduce the COD value to 1330.4 mg/L. As H_2_O_2_/Fe^2+^ or the reaction time increased, the oxidation efficiency gradually increased since visible light could only stimulate the reduction of Fe^3+^ to Fe^2+^ to maintain the catalytic cycle of the Fenton reaction, but the utilization of its light energy was limited, which decreased the **·**OH generation rate. The O_3_–Fenton+Vis method could reduce the COD value of the raw alkali slag solution to 894.9 mg/L, which was better than the degradation effect of the Vis–Fenton or O_3_–Fenton method. This was due to the strong oxidizing property of O_3_ and the synergistic effect of the Fenton reaction to increase the **·**OH yield. Visible light promoted the reduction of Fe^3+^ to reduce the generation of the iron sludge [32]. The UV–Fenton method was the most obvious method for reducing the COD value of the original solution since UV light could efficiently stimulate the reduction of Fe^3+^ to Fe^2+^ to accelerate Fenton oxidation. Additionally, the significantly increased **·**OH could have resulted in the direct photolysis of the organic matter, which further enhanced the COD removal effect.

**Figure 9:**
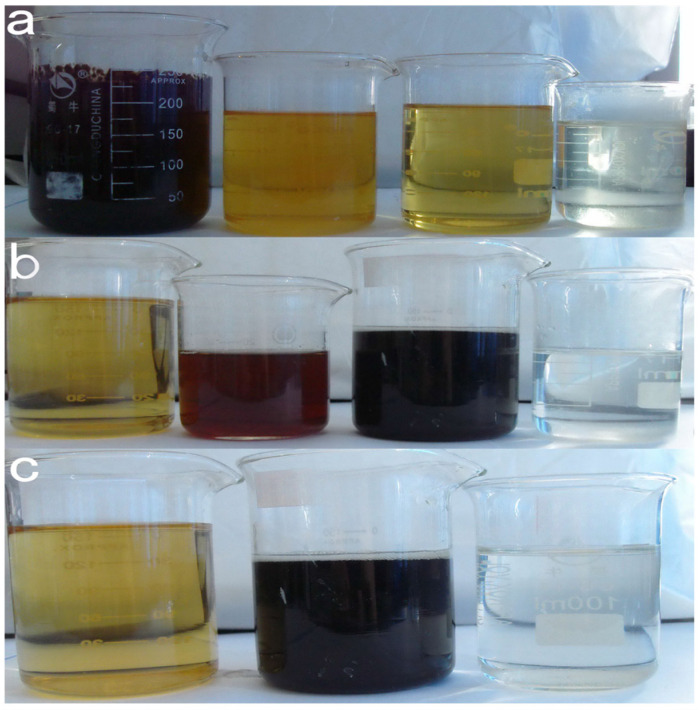
Photographs of three different composite Fenton oxidation treatments. (**a**) The Vis–Fenton method, (**b**) the O_3_–Fenton method, and (**c**) the UV–Fenton method.

After pretreatment, the alkali residue gradually changed its color from dark brown to colorless, and the suspended matter and sediment underwent the dynamic change in “aggregation-adsorption-oxidative degradation”. Primarily, the flocculation and adsorption stages physically separated the pollutants, while the Fenton oxidation completely degraded the organic matter through chemical reactions. As seen in Figure 9a, the original alkali residue solution showed the characteristic Fe^3+^ color after flocculation. Fe^3+^ was photo-reduced to Fe^2+^ with the introduction of visible light, and the solution gradually changed its color to light yellow. In the middle of the reaction, the solution changed its color from light green to turbid brown–yellow since a large **·**OH content was generated and the organic matter was effectively degraded. At the end of the reaction, the organic pollutants were effectively degraded, the solution had increased transparency, and the color turned light yellow or nearly colorless. With the oxidation of Fe^2+^ to Fe^3+^, a part of Fe^3+^ hydrolyzed to produce a reddish-brown flocculent precipitate (Fe(OH)_3_), which was seen in a small amount at the bottom. Visible light effectively promoted the reduction of Fe^3+^ (Fe^3+^ + H_2_O + hν → Fe^2+^ + **·**OH + H^+^), maintained Fe^2+^ recycling, and continuously generated **·**OH to accelerate the oxidation of organic matter.

As seen in Figure 9b, the initial solution was light-yellow colored after flocculation and adsorption of the alkali slag stock solution. Under the O_3_ irradiation, the test solution turned reddish-brown within 10 min (Fe^2+^ concentration increased). As the reaction continued, the organic matter broke the bond to generate small molecule intermediates (such as carboxylic acid), and the color of the solution gradually turned from dark green to light yellow to ultimately become colorless and transparent. The Fe^3+^ cycling rate was accelerated under the high energy provided by O_3_, which generated a small amount of Fe(OH)_3_ precipitation and fine particles to obtain a dispersed yellowish suspension, accompanied by tiny bubbles (O_2_ generated by the O_3_ decomposition) during the reaction. At the end of the reaction, the precipitates were partially dissolved, and the solution had a higher clarity than during the Vis–Fenton method. O_3_ directly oxidized the organic matter (O_3_ + organic matter → intermediate product), and simultaneously reacted with Fe^2+^ (O_3_ + Fe^2+^ → Fe^3+^ + O_3_^−^), which strengthened the free radical chain reaction and enhanced the reaction of O_3_ with Fe^3+^ to intensify the free radical chain reaction. When the alkali slag stock solution was passed through the UV light, it quickly turned dark brown as the reaction proceeded (UV and Fe^2+^ reacted to generate Fe^3+^ and **·**OH) due to the accumulation of organic oxidation, and ultimately turned lighter in color to finally turn colorless (Figure 9c). The strong oxidizing properties of the UV light rapidly generated Fe^3+^, resulting in numerous black-brown precipitates (FeO(OH) or Fe_2_O_3_). At the end of the reaction, the precipitates were collected at the bottom, and the upper layer of the solution was clarified. The UV light directly cracked H_2_O_2_ (H_2_O_2_ → 2 **·**OH) and stimulated the organic matter to produce the reactive intermediate, which synergistically worked with the Fenton reagent and remarkably enhanced the degradation effect.

**Figure 10:**
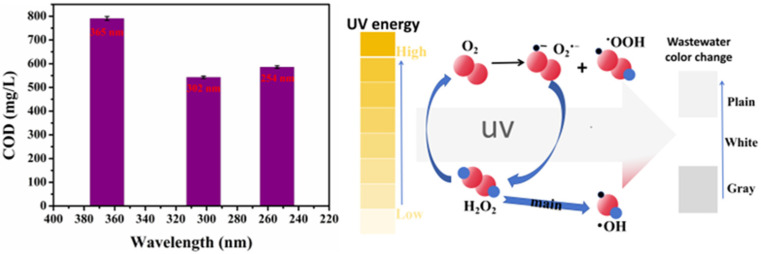
COD values after the stock solution was treated using the UV–Fenton method at different wavelengths.

**Figure 11:**
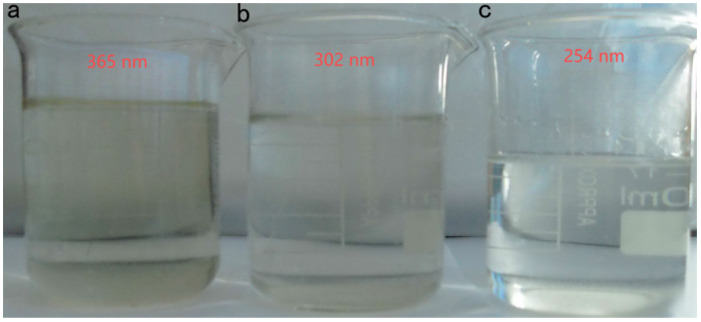
Photographs of the UV–Fenton-degraded test solution at different wavelengths. (**a**) 365 nm, (**b**) 302 nm, and (**c**) 254 nm.

Due to the different UV energies, the degradation effect on the original liquid had some differences (Figure 10). The treatment effect of different wavelengths of the UV-Fenton method on the stock solution was not directly proportional to the UV energy [33]. The COD reduction rate was slow after the UV intensity was increased to a certain degree. The 365 nm UV light was not enough to efficiently degrade the stock solution and could only oxidize part of it, which increased the COD value. The degradation ability of the stock solution increased as the UV energy increased. The COD value of the stock solution reached 543 mg/L when a 302 nm UV light was used for degradation. The degradation ability (TOC removal was 96.9%) of the stock solution was not enhanced as the UV energy continued to increase because the UV absorption capacity of the stock solution had reached its peak, and most of the UV light was not absorbed to produce more **·**OH. As seen in Figure 11, the stock solution degraded by a 302 nm UV–Fenton wavelength no longer contained flocculent precipitation, indicating that the UV–Fenton method at this wavelength could sufficiently degrade the stock solution. The high-energy UV light was efficient in degrading small organic molecules and could inhibit the generation of colored intermediates.

## 4. Conclusions

This study explored the use of Fenton oxidation for treating gasoline alkali residue stock liquids. Additionally, the influence of various factors on the degradation effect of stock liquids was analyzed. The gasoline alkali residue stock liquids during the production of Kashi refinery were experimentally studied to realize the optimal reaction conditions for treating this kind of stock liquid, and the primary conclusions are as follows:

(1) In the experiment for optimizing the H_2_O_2_/COD ratio, the H_2_O_2_ concentration was optimized. The **·**OH generation rate was found to be positively correlated with the H_2_O_2_ concentration. The COD and TOC removal first increased and then decreased with the increased H_2_O_2_ concentration. The **·**OH generation rate was higher for a higher H_2_O_2_ concentration. Excessive H_2_O_2_ could quickly oxidize Fe^2+^ to Fe^3+^ at the beginning of the reaction, and result in Fe^3+^-catalyzed oxidation, which consumed H_2_O_2_ and inhibited **·**OH generation to gradually increase the COD value. The COD value was reduced to the lowest when the H_2_O_2_ concentration was 1.4 mol/L.
(2) The orthogonal test to optimize the mass ratios of H_2_O_2_/Fe^2+^ found that the COD removal first increased and then decreased as the Fe^2+^ concentration increased. COD had the most obvious reduced value when m(H_2_O_2_):m(Fe^2+^) was 5:0.10.
(3) Effective pretreatment made the degradation effect of the Fenton oxidation treatment of the gasoline alkali residue more obvious. The UV–Fenton method had a more obvious degradation effect compared to the Vis–Fenton and O_3_–Fenton methods. Considering the production cost of this process, the highest H_2_O_2_ concentration was 1.0 mol/L and m(H_2_O_2_):m(Fe^2+^) was 5:0.10. A 302 nm UV–Fenton oxidation wavelength could be used to degrade the raw liquid to remove hazardous substances, and to treat gasoline alkali drugs in a low-investment, environment-friendly, and efficient way.

## Figures and Tables

**Table 1:** Optimization of the H_2_O_2_ concentration.

Sample Number	H_2_O_2_ Concentration (mol/L)	COD Values (mg/L)
1	0.2 mol/L	3816.5
2	0.4 mol/L	3252.1
3	0.6 mol/L	2702.4
4	0.8 mol/L	1975.3
5	1.0 mol/L	1639.7
6	1.2 mol/L	1489.2
7	1.4 mol/L	1321.6
8	1.6 mol/L	2943.2
9	1.8 mol/L	3424.9

**Table 2:** Optimization of the H_2_O_2_ and Fe^2+^ ratio in orthogonal test.

Sample Number	m(H_2_O_2_):m(Fe^2+^)	COD Values (mg/L)	Sample Number	m(H_2_O_2_):m(Fe^2+^)	COD Values (mg/L)
1	1:0.05	1454.7	16	3:0.20	1511.3
2	1:0.10	1360.2	17	3:0.25	1601.2
3	1:0.15	1815.4	18	3:0.30	1703.2
4	1:0.20	2042.3	19	4:0.05	1733.7
5	1:0.25	2404.2	20	4:0.10	1081.3
6	1:0.30	2529.8	21	4:0.15	1748.1
7	2:0.05	1505.4	22	4:0.20	1966.2
8	2:0.10	1334.9	23	4:0.25	2184.5
9	2:0.15	1646.3	24	4:0.30	2394.5
10	2:0.20	1797.1	25	5:0.05	1302.5
11	2:0.25	1914.1	26	5:0.10	810.7
12	2:0.30	2056.4	27	5:0.15	1215.1
13	3:0.05	2038.1	28	5:0.20	1425.1
14	3:0.10	1233.4	29	5:0.25	1499.4
15	3:0.15	1423.5	30	5:0.30	1582.8

**Table 3:** Standard electrode potentials for different oxidizing agents.

Oxidizing Agent	Chemical Reaction Equation	Redox Potential/V
**·**OH	**·**OH + H^+^ + e^−^ = H_2_O	28.10
O_3_	O_3_ + 2H^+^ + 2e^−^ = H_2_O + O_2_	22.07
H_2_O_2_	H_2_O_2_ + 2H^+^ + 2e^−^ = 2H_2_O	1.77
MnO_4_^−^	MnO_4_^−^ + 8H^+^ + 5e^−^ = Mn^2+^ + 4H_2_O	1.52
ClO_2_	ClO_2_ + 4H^+^ + 5e^−^ = Cl^−^ + 2H_2_O	1.51
Cl_2_	Cl_2_ + 2e^−^ = 2Cl^−^	1.36

## Data Availability

Data are contained within the article.
